# Fibroblast growth factor 23 level modulates the hepatocyte’s alpha-2-HS-glycoprotein transcription through the inflammatory pathway TNFα/NFκB

**DOI:** 10.3389/fmed.2022.1038638

**Published:** 2022-12-07

**Authors:** Deborah Mattinzoli, Min Li, Giuseppe Castellano, Masami Ikehata, Silvia Armelloni, Francesca Marta Elli, Paolo Molinari, Koji Tsugawa, Carlo Maria Alfieri, Piergiorgio Messa

**Affiliations:** ^1^Fondazione IRCCS Ca’ Granda Ospedale Maggiore Policlinico, Renal Research Laboratory, Milan, Italy; ^2^Fondazione IRCCS Ca’ Granda Ospedale Maggiore Policlinico, Unit of Nephrology, Dialysis and Renal Transplant, Milan, Italy; ^3^Department of Clinical Sciences and Community Health, University of Milan, Milan, Italy; ^4^Fondazione IRCCS Ca’ Granda Ospedale Maggiore Policlinico, Endocrinology Unit, Milan, Italy; ^5^Department of Pediatrics, Hirosaki University Hospital, Hirosaki, Japan

**Keywords:** AHSG, cardiovascular disease, chronic kidney disease, FGF23, NFκB

## Abstract

**Introduction:**

High serum levels of fibroblast growth factor 23 (FGF23) characterize chronic kidney disease (CKD) since its early stages and have been suggested to contribute to inflammation and cardiovascular disease. However, the mechanisms linking FGF23 with these pathological conditions remain still incompletely defined. The alpha-2-HS-glycoprotein (AHSG), a liver-produced anti-inflammatory cytokine, is highly modulated by inflammation itself, also through the TNFα/NFκB signaling pathway. In our previous study, we found that FGF23 modulates the production of AHSG in the liver in a bimodal way, with stimulation and inhibition at moderately and highly increased FGF23 concentrations, respectively.

**Methods:**

The present study, aiming to gain further insights into this bimodal behavior, was performed in hepatocyte human cells line (HepG2), using the following methods: immunochemistry, western blot, chromatin immunoprecipitation, fluorescence *in situ* hybridization (FISH), qRT-PCR, and gene SANGER sequencing.

**Results:**

We found that FGF23 at 400 pg/ml activates nuclear translocation of NFκB, possibly increasing AHSG transcription. At variance, at 1,200 pg/ml, FGF23 inactivates NFκB through the activation of two specific NFκB inhibitors (IκBα and NKIRAS2) and induces its detachment from the AHSG promoter, reducing AHSG transcription.

**Conclusion:**

These results add another piece to the puzzle of FGF23 involvement in the multifold interactions between CKD, inflammation, and cardiovascular disease, suggesting the involvement of the NFκB pathway, which might represent a potential therapeutic target in CKD.

## Introduction

Advanced chronic kidney disease (CKD) and mineral bone disorder (MBD) are often characterized by the presence of vascular calcification, also persisting after kidney recovery (1, 2). The osteocytes-derived fibroblast growth factor 23 (FGF23), principally implicated in phosphate and vitamin D regulation, is considered a promising and early CKD biomarker, which starts to increase from the beginning of renal diseases (3–5). Despite its compensative role, several extra-renal actions of FGF23 are strongly recognized and related to CV events: (a) stimulation of liver inflammatory cytokines production, (b) induction of cardiac myocytes hypertrophy, (c) harmful modulation of nitric oxide/oxygen-free radicals ratio causing endothelial dysfunction, atherosclerosis, and vascular calcification (6). Even though high FGF23 levels have been well recognized to be associated with most of the comorbidities of CKD patients, and in particular with cardiovascular disease (CVD), the pathogenic mechanisms by which FGF23 could induce these effects are far from being clear (7–9). It is well accepted that systemic inflammation contributes to CVD and mortality in CKD patients, and some recognized markers of inflammation [interleukin 1 and 6, tumor necrosis factor-alpha (TNFα), and c-reactive protein] are considered possible prognostic factors for CVD in a patient with CKD. Many recent studies strongly suggest that high FGF23 levels (10, 11) could be an inducer of inflammation, possibly stimulating TNFα and IL6 production by hepatocytes (12). In this context, some years ago, we found a strong relationship between FGF23 and alpha-2-HS-glycoprotein (AHSG) in the liver (13). The AHSG is a liver multifunctional protein, with two prominent roles linked respectively to its anti-inflammatory activity (acute phase protein) and its inhibitory effects on the calcification process (preventing calcium/phosphate complex deposition) (14–16). These properties propose AHSG as a potential biomarker and/or one of the most relevant risk factors for CVD in CKD (17, 18). In our previous paper, we explored the interactions between FGF23 and AHSG in the liver, and we found that: (1) FGF23 has a bimodal effect on AHSG, stimulating and inhibiting AHSG production at moderately elevated and highly elevated concentrations, respectively; (2) FGF23 protein can bind AHSG promoter; (3) FGF23 stimulates both TNFα and interleukin 6 liver production with a linear dose-dependent relationship (13). The main conclusions of our previous study were that FGF23, at very high levels, stimulates the inflammatory cytokines, which overwhelm the direct stimulating effect of FGF23 on AHSG production, acting as a trigger of some inflammatory pathways (19, 20). It’s well known that the biological effects of TNFα occur through the involvement of the nuclear factor kappa-light-chain-enhancer of activated B cells (NFκB). The NFκBs are a family of inducible transcription factors (TF), which regulate the expression of over 500 genes implicated in several inflammatory conditions (21–23). The modulation of the transcriptional activities of NFκB is finely regulated at many levels in both cytoplasm and nucleus by variable interactions of NFκB with IκB proteins family, which can alternatively induce activation or inhibition of NFκB, by mechanisms which have been described in detail in previous papers (24, 25). Moreover, NFκB is identified as a TF also for the AHSG gene (Softberry Inc., TFBS 3282, site ID: S03539) (26). In this study, we investigated the possible involvement of the NFκB signaling pathway, triggered by FGF23/TNFα, in the activation/deactivation of the AHSG transcription in liver cells.

## Materials and methods

### HepG2 cell culture

Human hepatocellular carcinoma line HepG2 (LGC Standards, Sesto San Giovanni, Italy) was cultured in IMDM + Glutamax^®^ with 10% FBS, 1% non-essential amino acids, and 1% penicillin/streptomycin (all from Gibco/Life Technologies, Milan, Italy). HepG2 cells were stimulated with human recombinant FGF23 (200–400–600–1,200 pg/ml) (Immunological Sciences, Roma, Italy) for 24 h. For FGFRs inhibition, FIIN 1 hydrochloride (1 μM, Tocris Bioscience, Bristol, UK) was administered for 6 h before adding FGF23.

### Immunocytochemistry and fluorescence *in situ* hybridization

HepG2 were fixed in cold acetone for 5 min or PFA for 10 min, permeabilized with 0.3% of Triton (Sigma-Aldrich, Milan, Italy) for 30 min and incubated with 1% of bovine serum blocking solution for 1 h. Immunocytochemistry was performed with the primary antibodies sheep anti-RelA/NFκB (R&D systems, Minneapolis, USA), mouse anti-IκBα (Santa Cruz), and rabbit anti-TNFα (Novus, Cambridge, UK) o/n. The secondary fluorescently labeled antibodies were used for 1 h RT: Alexa Fluor 488 donkey anti-sheep IgG, Alexa fluor 488 goat anti-mouse, and Alexa fluor 546 goat anti-rabbit (Invitrogen, Thermofisher, USA). The lack of staining demonstrated the specificity of Ab labeling after substituting the primary antibody with sheep IgG/mouse IgG1/rabbit IgG Isotype ctrl (Invitrogen). For fluorescence in situ hybridization (FISH), the Stellaris RNA protocol for adherent cells using the human AHSG target sequence was applied (Supplementary Table 1). Images were acquired by Zeiss AxioObserver microscope with Apotome system and recorded by AxioVision software 4.8. Human conditionally immortalized podocytes SV1 (HciPodo, University of Bristol, Bristol, UK) were used as negative ctrl. The nuclei were stained with DAPI (Sigma).

### Chromatin immunoprecipitation (CHIP), quantitative polymerase chain reaction, and Sanger sequencing

A chromatin immunoprecipitation (CHIP) of 10^6^ cells of HepG2 stimulated with FGF23 (200–1,200 pg/ml) was performed using the standard protocol (Merck GaA, Darmstadt, Germany) with CHIP-grade antibody mouse monoclonal anti-NFκB p65 (RelA) (ratio 5 ug/1 mg of protein) and anti-normal mouse IgG as negative ctrl (Merck GaA). PCR of human AHSG promoter was performed on 10% of total DNA (INPUT) as positive ctrl, on fragments immunoprecipitated (IP) with NFκB, and on anti-normal mouse IgG. The UCSC Gen Genome Browser identified human AHSG promoter primers (Supplementary Table 1) on GRCh37:3:186330112-186339707:1 by UCSC Genome Browser. The presence of PCR amplicon length 146 bp was resolved on 2% agarose gels containing 5 μl ethidium bromide in 0.5X Tris Borate EDTA buffer alongside a low molecular weight DNA ladder (Invitrogen) and acquired by GelDoc system (Bio-Rad, Milan, Italy). Amplification: PCR reagents (Roche, Monza, Italy) and steps 1: 6′ at 95°C, step 2: 35″ at 95°C, step 3: 35″ at 57, 5–60°C, step 4: 50″ at 72°C, step 5: from step 2 (×12), step 6: 35″ at 95°C, step 7: 35″ at 57°C, step 8: 50″ at 72°C, step 9: from step 6 (×23), step 10: 9’ at 72°C, step 11: 4°C. A Sanger sequencing verified the specificity of the fragment IP. A quantitative polymerase chain reaction (qRT PCR) of the fragments was performed, running the samples with iQ Sybr Green Supermix with human AHSG promoter primers on a MyIQ instrument (Bio-Rad). The melting curves confirmed the absence of primer-dimers (Supplementary Figure 1A).

### Gene expression

RNAs of HepG2 stimulated with FGF23 400–1,200 pg/ml with and without FIIN 1 hydrochloride were extracted using Trizol protocol followed by DNase (Invitrogen). cDNA was prepared from 300 ng RNA using the iScript Select cDNA Synthesis Kit and oligo(dt)20 primers (Bio-Rad). After the assessment of primer specificity, mRNA extracted was used to evaluate human FGF23, AHSG, TNFα, IκB kinase (IKK-β), and NFκB inhibitor interacting Ras-like 2 (NKIRAS2) (Supplementary Table 1). The absence of genomic DNA was verified on the original RNA (minus-reverse transcriptase) by qRT-PCR in triplicates. Data were normalized against human ribosomal protein L4 (RPL4). qRT-PCR was run with iQ Sybr Green Supermix on a MyIQ instrument, and data were analyzed by the IQ5 Software (Bio-Rad).

### Western blot

HepG2 at a density of 20,000 cells/cm^2^ treated with 400–1,200 pg/ml of FGF23 were lysed by complete Lysis-M kit (Roche) for cytoplasm and with a second lysis (60 μl of Lysis-M and 10 μl of NaCl 5 M) for the nuclear fractions. The protein lysates were separated on an SDS-PAGE and transferred by electroblotting on a PVDF membrane (Bio-Rad). After blocking, each membrane was incubated with the primary antibody sheep anti-RelA/NFκB 1: 500 (R&D) or anti-TNFα 1:250 (Novus) followed by the HRP-conjugated secondary antibodies 1:200. The products were identified by chemiluminescence (BM Chemiluminescence Western Blotting Kit, Roche). Loading ctrl was conducted with antibodies directed rabbit anti-cofilin 1: 10,000 (SIGMA) and against anti-Histone H3 for Nuclear loading ctrl 1: 1,000 (not shown, Abcam). The images were acquired/analyzed by the Chemidoc XRS instrument and Quantity One software (Bio-Rad).

### Tumor necrosis factor-alpha silencing with FGF23 addition

For TNFα silencing, 20,000 HepG2 cells/cm^2^ were transfected with 40 pmol siRNA using Lipofectamine 2000 (Invitrogen) for 30 h. Four commercially available siRNA complementary to TNFα mRNA were used (Dharmacon, USA) (Supplementary Table 1). A negative control non-targeting siRNA pool (Dharmacon) (Supplementary Table 1) along with lipofectamine 2000 to avoid the possibility of a non-specific target of the siRNA was applied. The FGF23 recombinant was added 6 h after the transfection.

### Statistical analyses

Experiments were conducted on at least three replicates per condition and time point. Data were expressed as mean ± standard deviation; Student’s *t*-test two tails analysis was used when two groups of data were compared and *p* < 0.05 was considered significant. For qRT-PCR, relative RNA abundance was determined using the comparative Ct method. Fold change error bars represent the standard deviation of the fold change. For IκBα and TNFα immunostaining quantification, ImageJ program was used and *n* = 9 and 20 cells respectively were counted. The CHIP-qRT-PCR data were analyzed following the Thermofisher scientific method and with an ANOVA test.

## Results

### Effect of FGF23 addition on FGF23, tumor necrosis factor-alpha, and alpha-2-HS-glycoprotein expression

**FIGURE 1:**
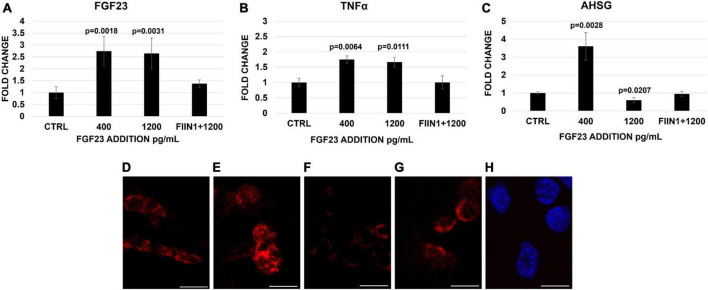
Changes of FGF23 **(A)**, TNFα **(B)**, and AHSG **(C)** mRNA expression 24 h after the addition of FGF23 at 400 pg/ml and then at 1,200 pg/ml without and with FIIN 1 hydrochloride (FIIN1) in HepG2. FISH of AHSG mRNA in the ctrl **(D)** and 24 h after the addition of FGF23 at 400 pg/ml **(E)** and 1,200 pg/ml without **(F)** and with FIIN1 **(G)** in HepG2 and podocytes **(H)** as negative ctrl. *N* = 4 **(A–C)**. Scale bar 50 μm.

To confirm our previous results, the expression of FGF23, TNFα, and AHSG was examined in HepG2 treated with increasing FGF23 concentrations, with and without FIIN 1 hydrochloride (FGFrs receptor blockade). The mRNA expression of FGF23 and TNFα increased after either 400 or 1,200 pg/ml addition of FGF23, compared to the ctrl; however, the addition of the irreversible FGFR inhibitor FIIN 1 hydrochloride to 1,200 pg/ml of FGF23 abolished the changes of mRNA of the markers (Figures 1A,B). A significant increase of TNFα protein expression after FGF23 addition was also observed (Supplementary Figures 2C–E,I). The increase of the AHSG mRNA expression after the addition of 400 pg/ml of FGF23 and its reduction after the exposure to 1,200 pg/ml was also confirmed by FISH of AHSG mRNA (Figures 1C–H). No change was observed with the blockade of the FGFRs, followed by the addition of the highest concentration of FGF23 (Figures 1C–H).

### NFκB distribution in the HepG2

**FIGURE 2:**
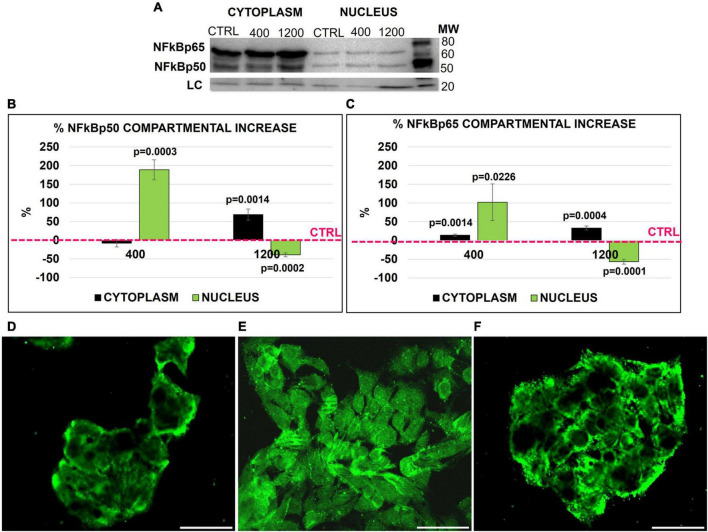
NFκB (p50 and p65) Western blot (WB) in the nucleus and cytoplasm of HepG2 in the ctrl and 24 h after adding 400 and 1,200 pg/ml of FGF23 **(A)**. NFK Bp65/p50 compartmental protein quantification. Cytoplasm and nucleus % increase normalized by cofilin loading control and against the respective compartmental ctrl **(B,C)**. Immunostaining of NFκB in the ctrl **(D)** and 24 h after the addition of 400 **(E)** and 1,200 pg/ml **(F)** of FGF23. *N* = 3 **(B,C)**, Scale bar 50 μm.

Therefore, we investigated the quantitative and percentage changes of NFκB subunits p50 and p65 in both the nucleus and cytoplasm of HepG2 cells after FGF23 addition. Both subunits were present in the cytoplasm and nucleus in basal conditions (Figure 2A). The addition of FGF23 was associated with an increase in the amounts of the two proteins in both cytosol and nuclear compartments; however, the kinetics of subunit increase was quite different in each compartment, depending on the added dose of FGF23. In fact, after the addition of 400 pg/ml of FGF23, a marked nuclear increase of both subunits was observed only in the nuclear compartment, while the addition of 1,200 pg/ml was associated with a mild percentage increase in the cytoplasm and even a decrease in the nucleus of both subunits (Figures 2B,C). From here onward, we limited our experiments to check for the p65 subunit only since it contains the C-terminal transcriptional activation domain (27). The IF highlighted the different compartmental changes of NFκB p65, which was increased in the nucleus after the addition of 400 pg/ml of FGF23, with almost complete exhaustion of this finding at 1,200 pg/ml (Figures 2D–F).

### IκBα distribution in the nucleus and cytoplasm and IKK-β expression

**FIGURE 3:**
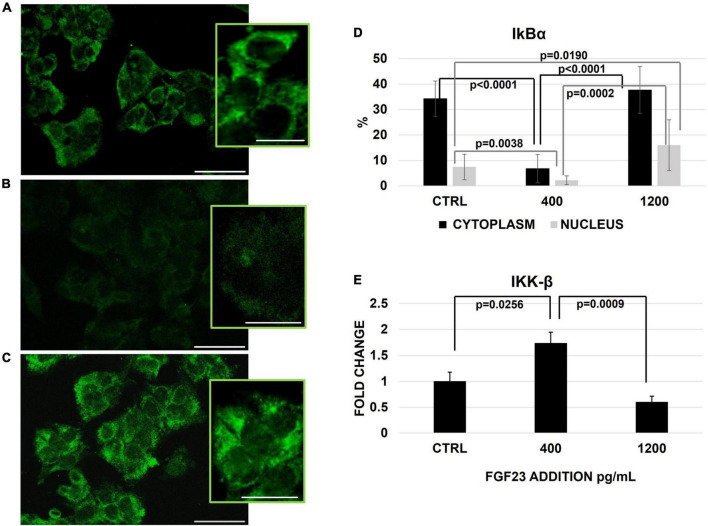
Immunostaining of IκBα in the ctrl **(A)** and 24 h after the addition of 400 **(B)** and 1,200 **(C)** pg/ml of FGF23 visualized by FITC and its staining quantification with ImageJ program in the nucleus and cytoplasm. Changes of IKK-β mRNA expression 24 h after the addition of FGF23 at 400 pg/ml and at 1,200 pg/ml. *N* = 11 **(D)**, *N* = 6 **(E)**. Scale bar 50 μm.

We then explored if these changes are due to a translocation of NFκB between the cytosol and nuclear compartments. Since IκBα activation/degradation could modulate the NFκB shuttling, under the control of the IKK-β specific kinase inducing IkBα degradation, the behavior of these two factors was explored at each experimental condition. In basal conditions, IκBα protein was well represented in both cytoplasm and nucleus, though it was by far higher in the former compartment; at 400 pg/ml of FGF23, IκBα protein markedly decreased in both compartments, with a recovery at 1,200 pg/ml. Of note, the increase of IκBα observed at 1,200 pg/ml, was even more evident in the nuclear compartment than in the cytosol, as compared with control conditions (Figures 3A–D). The IKK-β mRNA expression and activation increased significantly only at 400 pg/ml (Figure 3E).

### Detection and quantification of% of alpha-2-HS-glycoprotein promoter linked to NFκB and NKIRAS expression

**FIGURE 4:**
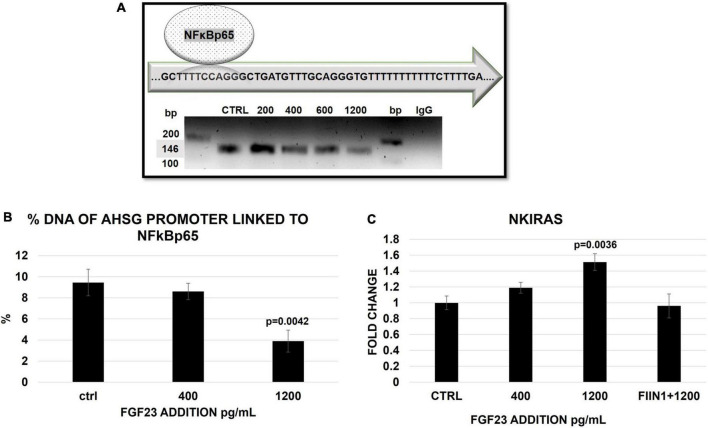
Semi-quantitative PCR of AHSG promoter (146 bp) in HepG2 IP with ab anti-NFκB in the ctrl and 24 h after a scalar addition of FGF23 (200–1,200) **(A)**. The false positivity was excluded by the negative ctrl normal mouse IgG **(A)**, while the correctness of the PCR was confirmed by the input (10% DNA) in the sample ctrl, 400 and 1,200 pg/ml (Supplementary Figure 1B). Percentage of AHSG promoter linked to NFκB 24 h after a scalar addition (400–1200 pg/ml) of FGF23. Changes of NKIRAS2 mRNA expression 24 h after the addition of FGF23 at 400 pg/ml and at 1,200 pg/ml without and with FIIN 1 hydrochloride (FIIN1) in HepG2. *N* = 3 **(B)**, *N* = 6 **(C)**.

We then tested whether these FGF23-related changes in the cytosol/nuclear distribution of NFκB were involved in the transcription-regulation of the AHSG promoter. Confirming results from previous studies, NFκB co-precipitated with the specific band (146 bp) of the AHSG promoter in the ctrl cells (HepG2); the Sanger sequencing confirmed that the chromatin fragment immune-precipitated with the antibody overlaps the sequence of AHSG promoter on chromosome GRCh37:3:186330696–186330831. The link between NFκB and AHSG promoter persisted at any level of FGF23 exposure (from 200 to 1,200 pg/ml), although a trend to a decrease at higher FGF23 exposures was evident (Figure 4A). Then, to better qualify this trend as a reduction, we checked for the amount of AHSG promoter bound to NFκB by the qRT-PCR of the fragments at the most relevant experimental concentrations of FGF23 (see section “Materials and methods”). While no reduction of the promoter linked NFκB was detected at 400 pg/ml of added FGF23, a decrease occurred at 1,200 pg/ml (Figure 4B). To explore whether changes of Ras-like 2 (NKIRAS), an inhibitor of the NFK B in the nuclear compartment, were also involved, we examined its expression under the same experimental conditions; these assessments were repeated after blocking FGF23 receptors. The NKIRAS expression significantly increased at 1,200 pg/ml but not at 400 pg/ml of FGF23; this change was entirely abolished by FGF23 receptor blocking (Figure 4C).

### Effect of FGF23 addition after the tumor necrosis factor-alpha silencing

**FIGURE 5:**
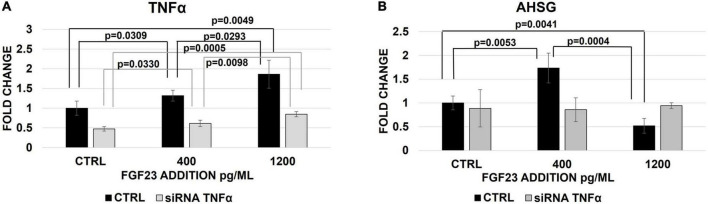
Changes of TNFα **(A)** and AHSG **(B)** mRNA expression 30 h after the treatment with non-targeted siRNA (CTRL) or TNFα silencing and 24 h after the exposure to 400 and 1,200 pg/ml of FGF23 in HepG2. *N* = 4.

To better qualifying the role of TNFα in the FGF23-mediated changes of AHSG, we analyzed the expression of AHSG mRNA after the TNFα silencing followed by the addition of FGF23. FGF23 addition was associated with a scalar increase of TNFα mRNA (Figure 5A) and protein expression (Supplementary Figure 2), as expected, and after the silencing, although to a lesser extent and The AHSG mRNA increased and decreased after the addition of 400 pg/ml and 1,200 pg/ml of FGF23, respectively, and this bimodal behavior of AHSG was abolished after the silencing (Figure 5B).

## Discussion and conclusion

Recently, we found that FGF23 has a bimodal modulatory effect on AHSG in liver cells, stimulating or inhibiting AHSG production at moderately high or highly elevated FGF23 concentrations, respectively, associated with a monomodal progressive stimulation of TNFα (13). It has already been reported that high FGF23 promotes inflammation in the liver, the main site of AHSG production, in a CKD animal model (12). In this background, we aimed to explore the mechanisms underlying FGF23 regulatory effects on AHSG and inflammatory cytokines (12, 13, 28, 29). Among inflammatory cytokines, we have chosen to study TNFα, whose production in liver cells has been already reported to be stimulated by FGF23 (12, 13, 30). The NFκB pathway was studied since it represents the classic pro-inflammatory pathway strongly related to the TNFα cascade, and it also constitutes a TF for the AHSG promoter (26, 31–33).

We decided to use two concentrations of FGF23 (400 and 1,200 pg/ml) since we previously demonstrated that they represent the doses inducing both the maximal upregulation and downregulation of AHSG, respectively (13). In the first step, after reconfirming the bimodal and monomodal effects of FGF23 on AHSG and TNFα, we demonstrated that these effects are entirely mediated by one or more of the four FGF23 receptors since their blockage completely abolished the above described FGF23 effects on both cytokines. Since the FGF23 receptor expressed in liver cells is mostly FGFR4, further research should address whether one or all of them are involved in this signaling pathway (34, 35).

It is well recognized that the pro-inflammatory effects of TNFα occur through the involvement of the NFκB, which is also involved in activating the AHSG promoter gene. Since it is also known that NFκB is strictly controlled by the inhibitory protein IκBα, we explored the behavior of the NFκB/IκBα pathway.

NFκB, composed of the dimer p50-p65, is present in the cytoplasm bound to the inhibitory protein IκBα, which prevents its entering into the nucleus, where, once entered, it acts binding the target DNA sequences (36). The inflammatory signal, such as TNFα activation, induces the IK Bα phosphorylation by activation of IκB kinase (IKK), which is followed by IKBα degradation, letting free NFκB move into the nuclear compartment (31, 37, 38).

For these reasons, we quantified the nuclear and cytoplasmatic changes of the NFκB/IKBα pathway in HepG2 after 400 and 1,200 pg/ml of FGF23 addition compared to the respective controls. The results highlighted that at 400 pg/ml, both the components of NFκB dimer, p50 and p65, were highly increased in the nucleus, while, at 1,200 pg/ml, their increase was evident primarily in the cytoplasm, possibly suggesting a coming back in a resting state.

The above behavior of the NFκB components was consistent with that of IκBα and IKK. We observed a striking reduction of IkBα only at 400 pg/ml of FGF23, the condition under which we have also obtained the overexpression of the kinase IKK responsible for its degradation. Conversely, in the ctrl and at 1200 pg/ml, no activation of the IKK occurred, and the IκBα was evident in the cytoplasm. This bimodal behavior of IKK is not counterintuitive and deserves further investigation.

Given that NFκB behaves as a TF for the AHSG gene promoter, after confirming the link between NFκB and the AHSG promoter, we examined whether this bond is affected by a scalar FGF23 addition. The progressive reduction of the NFκB/AHSG promoter bond following the stepwise increase of FGF23 concentration suggests that a significant detachment of NFκB from the AHSG promoter might partially explain the reduction of AHSG production observed at 1,200 pg/ml of FGF23. To reinforce our hypothesis, we also analyzed the nuclear NFK B inhibitor NKIRAS2, which suppresses the phosphorylation of the NFκB-p65 subunit (at serine 276), inhibiting its interaction with the p300-CBP coactivator necessary for the link of NFκB to the AHSG promoter (39, 40). We observed an activation of the NKIRAS2 at 1,200 pg/ml of FGF23, confirming the NFκB deactivation and detachment from the AHSG promoter. Finally, the silencing of TNFα abolished the bimodal effect of FGF23 on AHSG, validating its intermediary role in the mechanism.

Despite the novelties presented in the present work, some limitations occur. First, confirming our findings in an animal model could be suitable. It will be the issue of our future research. The principal aim of this research was to complete the results obtained in our previous study and published where an “*in vitro* system” is essential to provide a controlled environment for analyzing this pathway signaling. An *in vivo* model requires precise and controlled planning both in the disease model and experiments and deserves specific and dedicated publication. Secondly, the data will be then confirmed by human plasma ELISA quantification.

In conclusion, the present study suggests that NFκB could be the link for explaining at least in part the mechanisms underlying the complex interplay between FGF23, TNFα, and the modulation of AHSG transcription. NFκB, which is strictly dependent on TNFα, behaves as the TF of the AHSG promoter. We found that high concentrations of FGF23 increase the detachment of NFκB from the AHSG promoter through IκBα and NKIRAS2 activation. Though our results provide clear evidence of AHSG transcription modulation dependence from FGFRs and FGF23 concentrations, several open issues ensue from these initial results: (i) specify the TNFα functional role in the mechanism and (ii) clarify the enhancing effect of moderately high FGF23 levels on AHSG production. Future studies are expected to give more insight into these issues.

## Data availability statement

The datasets presented in this study can be found in online repositories: doi: 10.6084/m9.figshare.20980939. The names of the repository/repositories and accession number(s) can be found in the article/Supplementary material.

## Author contributions

DM and ML: research and study design, data analysis, interpretation, writing – original draft, and supervision and final approval of the version to be published. GC, MI, FE, and SA: investigation. PMo and KT: statistical analysis. CA and PMe: writing – review and editing. All authors contributed to important intellectual content during manuscript drafting or revision, agreed to be accountable for all aspects of the work to ensure that questions related to the accuracy or integrity of any portion of the work are appropriately investigated and resolved, and provided approval for publication of the content.
