# Supplementary material for: Fibroblast growth factor 23 level modulates the hepatocyte’s alpha-2-HS-glycoprotein transcription through the inflammatory pathway TNFα/NFκB
Source: Front Med (Lausanne). 2022 Dec 7;9:1038638. doi: 10.3389/fmed.2022.1038638 (PMC9769965; doi:10.3389/fmed.2022.1038638)
Supplement: Supplementary file 1 [file Data_Sheet_1.pdf]

Supplementary Material

Supplementary Table 1: Primers and siRNA target sequence.

**HUMAN**			
**GENE**	**FORWARD PRIMERS**	**REVERSE PRIMERS**	**NCBI**
	**5’ → 3’**	**5’ → 3’**	
FGF23	CCT TGT GCC TCT CCT CTT TAT C	GAT TTC CTC TTC CCT ACA CCT TC	NM_020638.3
AHSG	GCA GCT CTG GTG GCT ATA GA	CTT CGA CAG CAT GCT CCT TC	NM_001622.2
TNFα	AGG ACC AGC TAA GAG GGA GA	CCC GGA TCA TGC TTT CAG TG	NM_000594.3
NKIRAS2	CCT CAC TGG TTG AGA TGG AAT AG	GAG AAA CTG ATG CCC AGA GAG	NM_001144929.2
IKK-β	AAA AGT GCG GCA GAA GAG TG	ATG TCA TCC AGG GCC TTG AA	NM_001190720.3
RPL4	CGA GCA CCA CGC AAG AAG ATC CA	AAT GGT GTT CCG GCG CAT GGT	NM_000968.3
**RNA FISH**	**FORWARD PRIMERS**	**REVERSE PRIMERS**	**NCBI**
	**5’ → 3’**	**5’ → 3’**	
AHSG	GCA GCT CTG GTG GCT ATA GA	CTT CGA CAG CAT GCT CCT TC	NM_001622.2
**GENE**	**FORWARD PRIMERS**	**REVERSE PRIMERS**	**UCSC Genome Browser**
AHSG promoter	CAG CGA TGT CCT AAC CTG TTT	GAG GGT GGT GGG CTT TAT TT	
**TARGET SEQUENCE**	**NON-TARGETING ((DHARMACON)**		
FIRST	UGG UUU ACA UGU CGA CUA A		
SECOND	UGG UUU ACA UGU UGU GUG A		
THIRD	UGG UUU ACA UGU UUU CUG A		
FOURTH	UGG UUU ACA UGU UUU CCU A		
**TARGET SEQUENCE**	**TNFα SiRNA (DHARMACON)**		
FIRST	GCC CGA CUA UCU CGA CUU U		
SECOND	GCG UGG AGC UGA GAG AUA A		
THIRD	UGA CAA GCC UGU AGC CCA U		
FOURTH	CCA GGG ACC UCU CUC UAA U		

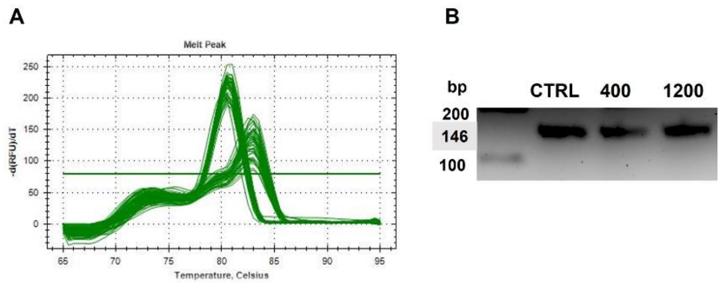

Supplementary Figure 1: The representation of the Melt curve confirming the absence of primer dimer and the purity of the reaction **(A)**. Correctness of the AHSH promoter PCR confirmed by the input (10% DNA) in the sample ctrl, 400, and 1200pg/mL IP with NFκB **(B)**.

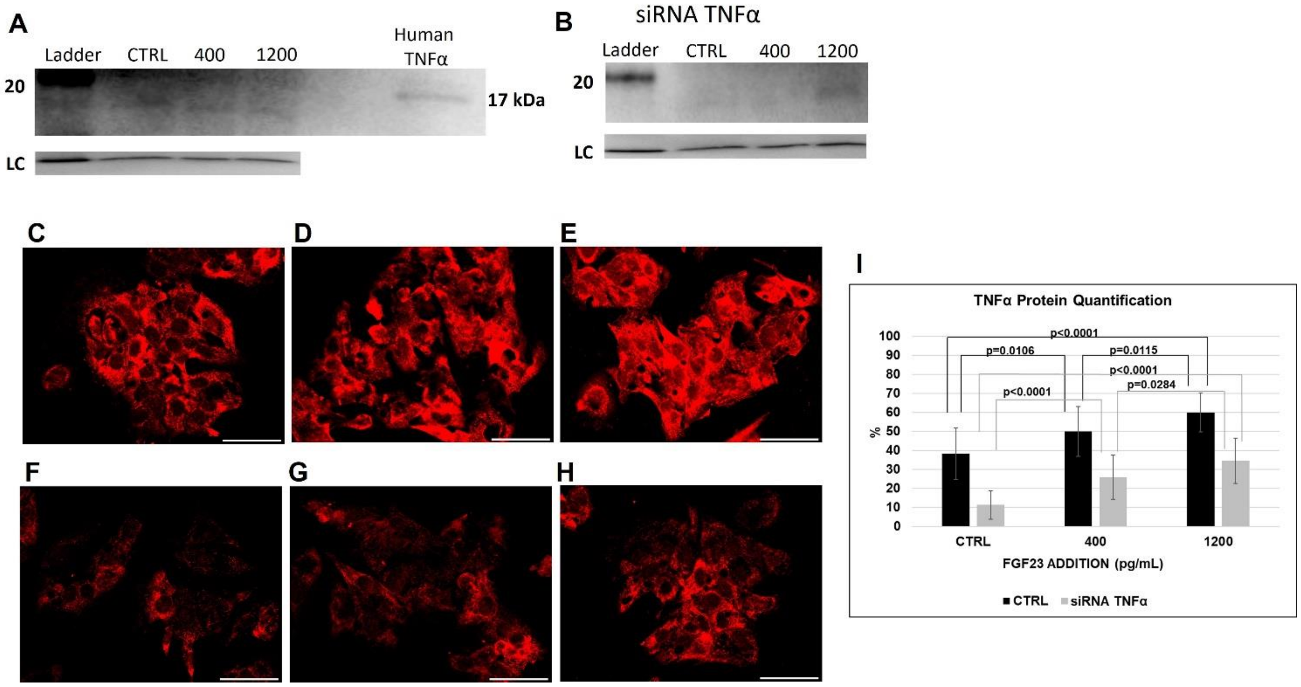

Supplementary Figure 2: WB of TNFα 30h after the treatment with non-targeted siRNA **(A)** or siRNA TNFα **(B)** and 24h after the scalar FGF23 addition. TNFα Recombinant Positive control at 17kDa. TNFα immunostaining in the ctrl **(C)**, and 24h after the FGF23 addition at 400 **(D)**, and 1200 pg/mL **(E)** in HepG2. TNFα immunostaining 30h after the TNFα silencing followed by exposure to 0 pg **(F)**, 400pg/mL **(G)**, and 1200 pg/mL of FGF23 **(H)**. The semi-quantitative analysis of TNFα protein expression with IMAGE J program **(I)**. N=20 **(I)**. Scale bar 50μm.
